# Supplementary material for: Splice-Junction-Based Mapping of Alternative Isoforms in the Human Proteome
Source: Cell Rep. Author manuscript; Available in PMC 2020 Jan 15. (PMC6961840; doi:10.1016/j.celrep.2019.11.026)

A

**sp|Q96DB9|FXYD5_HUMAN|ENSG00000089327|MXE1|103|chr19|35158400|35160801|+2|r25|T1**
**AGFHEDDPFFYDEHTLR q value: 0.0027865 Tr_novel:TRUE RefSeq_Novel:TRUE**
**Search result spec prec mz: 525.2356 Actual spec prec mz: 525.2356**
**Fragments matched per AA: 1.24 Proportion of top 20 peaks matched: 0.35**

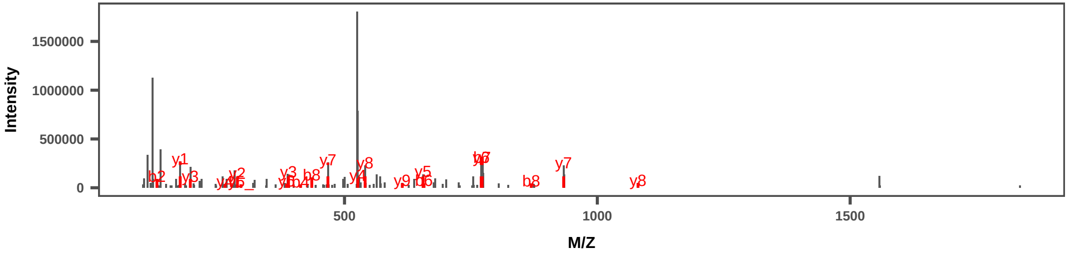


B

**Scatterplot of predicted elution time**
**Fitting R2: 0.852**
**Novel peptide residual Z score: −1.83**
**Number of peptides: 6934**

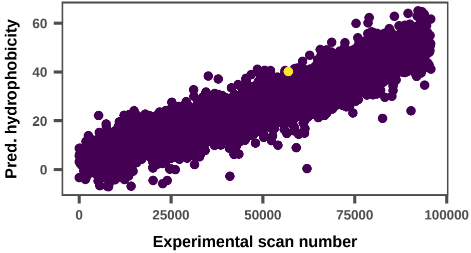


C

**Distributions of residuals from best−fit line**
**of predicted RT vs Expt. scan number**
**Line: Z score of novel peptide**
**Z: −1.83**

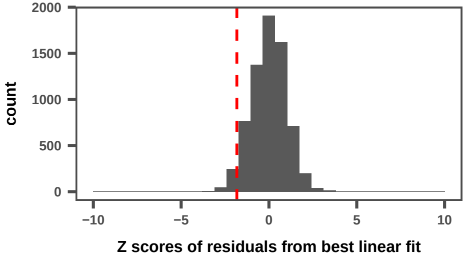

Supplement: 2 [file NIHMS1546469-supplement-2.zip › DF1/PXD006675/CardiacFibroblast/CardiacFibroblast_23_FXYD5_AGFHEDDPFFYDEHTLR.pdf]
